# Supplementary material for: Potential Regional Pollination Services of Spodoptera litura (Lepidoptera: Noctuidae) Migrants as Evidenced by the Identification of Attached Pollen
Source: Plants (Basel). 2024 Dec 11;13(24):3467. doi: 10.3390/plants13243467 (PMC11728779; doi:10.3390/plants13243467)
Supplement: Supplementary file 1 [file plants-13-03467-s001.zip › plants-3316760-supplementary.pdf]

Supplementary Materials:

Table S1. Pollen source plants identified at the species level visited by *S. litura* migrants from 2018 to 2021

Pollen Num.	Species	Taxonomic Status	Plant type	Florescence	Distribution range in China
1	*Cynanchum rostellatum*	Apocynaceae, Cynanchum	Herbaccous plant	Jul.-Aug.	Northeast China, North China, East China, Gansu, Shaanxi, Guizhou, Hubei
2	*Cynanchum chinense*	Apocynacea, Cynanchum	Herbaccous plant	Jun.-Aug.	Liaoning, Beijing, Tianjin, Hebei, Inner Mongolia, Shandong, Shaanxi, Ningxia, Gansu, Jiangsu, Zhejiang
3	*Vincetoxicum atratum*	Apocynacea, Vincetoxicum	Herbaccous plant Herbaccous plant	Apr.-Aug.	Central and eastern China
4	*Albizia julibrissin*	Fabaceae, Albizia	Woody plant	Jun.-Jul.	Distributed across China
5	*Lycium chinense*	Solanaceae, Lycium	Woody plant	May-Sept.	Distributed across China
6	*Atractylodes lancea*	Asteraceae, Atractylodes	Herbaccous plant	Jun.-Oct.	Northeast China, North China, East China, Central China, Sichuan Province
7	*Artemisia argyi*	Asteraceae, Artemisia	Herbaccous plant	Jul.-Oct.	Distributed across China
8	*Chrysanthemum lavandulifolium*	Asteraceae, Chrysanthemum	Herbaccous plant	Jun.-Aug.	Heilongjiang, Jilin, Liaoning, Inner Mongolia, Hebei, Shaanxi, Gansu, Henan, Hubei, Huan, Yunnan, Sichuan
9	*Chrysanthemum zawadzkii*	Asteraceae, Chrysanthemum	Herbaccous plant	Jul.-Sept.	Inner Mongolia, Hebei, Shanxi, Shaanxi, Gansu, Anhui, and northeast provinces

10	*Adenophora trachelioides*	Campanulaceae, Adenophora	Herbaccous plant	Jul.-Sept.	Jiangsu, Zhejiang, Anhui, Shandong, Chongqing, north and northeast provinces
11	*Humulus scandens*	Cannabaceae, Humulus	Herbaccous plant	Mar.-Sept.	Distributed across China, except Xinjiang and Qinghai
12	*Eleusine indica*	Poaceae, Eleusine	Herbaccous plant	Jun.-Oct.	Distributed across China
13	*Tamarix chinensis*	Tamaricaceae, Tamarix	Woody plant	Apr.-Sept.	Provinces from eastern to southwestern China
14	*Suaeda glauca*	Amaranthaceae, Suaeda	Herbaccous plant	Jul.-Sept.	Heilongjiang, Inner Mongolia, Jiangsu, Zhejiang, north and northwestern provinces
15	*Cuscuta japonica*	Convolvulaceae, Cuscuta	Herbaccous plant	Aug.	Distributed across China
16	*Flueggea suffruticosa*	Phyllanthaceae, Flueggea	Woody plant	Mar.-Aug.	Distributed across China, except the northwest provinces

Table S2. Date of *Spodoptera litura* migrants carrying *Chrysanthemum zawadskii* and *Adenophora trachelioides* pollens, also chosen for trajectory simulation.

Pollen species	Date of trapped moths	Pollen species	Date of trapped moths
	2018/10/1		2018/8/27
	2019/9/30		2019/9/12
	2019/10/1		2019/9/21
	2019/10/3		2019/9/30
	2019/10/8	*Adenophora trachelioides*	2019/10/1
	2019/10/9		2019/10/3
	2019/10/11		2019/10/11
	2019/10/18		2020/9/18
Chrysanthemum zawadskii	2019/10/19	-	
	2019/10/20	-	
	2019/10/22	-	
	2019/10/23	-	
	2020/9/23	-	
	2020/10/5	-	
	2020/10/11	-	
	2020/10/17	-	
	2020/10/20	-	
	2020/10/24	-	

Table S3. Parameters setting of micropipette puller for self-made glass needle

Heat	Pull	Vel.	Delay	Pressure	Ramp
570	0	25	1	450	562

Table S4. Primers for amplification of pollen genes fragment

Regions	Primer name	Sequences (5′-3′)	References
rbcL	rbcl-a-F	ATGTCACCACAAACAGAAAC	[1]
	rbcl-a-R	TCGCATGTACCTGCAGTAGC	
	rbcl-b-F	ATGTCACCACAAACAGAAAC	[2]
	rbcl-b-R	GAAACGGTCTCTCCAACGCAT	
ITS	ITS-F	GACTCTCGGCAACGGATATC	[3]
	ITS4-R	TCCTCCGCTTATTGATATGC	[4]
*psb*A-*trn*H	trnH-F	CGAAATCGGTAGACGCTACG	[5]
	trnH-R	ATTTGAACTGGTGACACGAG	

References:

1. Fay, M.F.; Swensen, S.M.; Chase, M.W. Taxonomic Affinities of Medusagyne oppositifolia (Medusagynaceae). *Kew Bulletin* **1997**, *52*. doi: 10.2307/4117844.

2. Fazekas, A.J.; Burgess, K.S.; Kesanakurti, P.R.; Graham, S.W.; Newmaster, S.G.; Husband, B.C.; Percy, D.M.; Hajibabaei, M.; Barrett, S.C. Multiple multilocus DNA barcodes from the plastid genome discriminate plant species equally well. *PLoS ONE* **2008**, *3*, e2802. doi: 10.1371/journal.pone.0002802.
3. Amaral, L.A.N.; Ait Baamrane, M.A.; Shehzad, W.; Ouhammou, A.; Abbad, A.; Naimi, M.; Coissac, E.; Taberlet, P.; Znari, M. Assessment of the food habits of the Moroccan Dorcas gazelle in M'Sabih Talaa, west central Morocco, using the *trn*L approach. *PLoS ONE* **2012**, *7*. doi: 10.1371/journal.pone.0035643.
4. White, T.J.; Bruns, T.D.; Lee, S.B.; Taylor, J.W. Amplification and direct sequencing of fungal ribosomal RNA genes for phylogenetics. In *PCR Protocols: A Guide to Methods and Applications*; Academic Press, Inc.: New York, 1990; pp. 315-322. 0968-0004.
5. Taberlet, P.; Gielly, L.; Pautou, G.; Bouvet, J. Universal primers for amplification of three non-coding regions of chloroplast DNA. *Plant Molecular Biology* **1991**, *17*, 1105-1109. doi: 10.1007/BF00037152
